# Associations Between Physiological Characteristics and Physical Performance in Amputee Soccer Players: A Cross-Sectional Study

**DOI:** 10.3390/medicina62071346

**Published:** 2026-07-12

**Authors:** Mert Ilhan, Fatih Erbahceci

**Affiliations:** 1 Department of Physiotherapy and Rehabilitation, Faculty of Health Sciences, Uskudar University, Istanbul 34662, Turkey; 2 Graduate School of Health Sciences, Hacettepe University, Ankara 06100, Turkey; 3 Faculty of Physical Therapy and Rehabilitation, Hacettepe University, Ankara 06100, Turkey

**Keywords:** amputees, athletic performance, core stability, muscle strength, soccer

## Abstract

*Background and Objectives*: Amputee soccer places exceptional demands on lumbopelvic stability and upper extremity capacity due to unilateral lower limb amputation and forearm crutch use. This cross-sectional study examined associations between core stabilization, core endurance, respiratory muscle strength, isokinetic knee muscle strength, and dominant handgrip strength with physical performance in amputee soccer players, and examined which physiological variables were most strongly associated with performance. *Materials and Methods*: Fifteen male amputee soccer players (age: 27.4 ± 5.6 years; BMI: 20.8 ± 1.4 kg/m^2^) competing in the Turkish Amputee Football Super League were included. Core stabilization was assessed by the pressure biofeedback unit (PBU), core endurance by the McGill Core Endurance Battery, respiratory muscle strength by maximal inspiratory and expiratory pressures (MIP and MEP), isokinetic knee muscle strength at 60°/s, 180°/s, and 240°/s using the IsoMed 2000 dynamometer, and dominant handgrip strength by a hydraulic hand dynamometer. Physical performance outcomes were single-leg triple hop test (THT) distance, 10-m sprint time, and Closed Kinetic Chain Upper Extremity Stability Test (CKCUEST) score. *Results*: PBU score was negatively correlated with THT distance (r = −0.928, *p* < 0.001) and 10-m sprint time (r = −0.580, *p* = 0.023). McGill total score was correlated with THT distance (r = 0.561, *p* = 0.030), and dominant handgrip strength with CKCUEST score (r = 0.634, *p* = 0.011). No significant associations were found for respiratory muscle strength or isokinetic knee muscle strength. Separate regression models showed that PBU score explained variance in THT distance (R^2^ = 0.861, *p* < 0.001) and 10-m sprint time (R^2^ = 0.336, *p* = 0.023); McGill total score explained variance in THT distance (R^2^ = 0.315, *p* = 0.030). *Conclusions*: Core stabilization and endurance appear to be primary factors associated with physical performance in amputee soccer and warrant targeted assessment and training.

## 1. Introduction

Amputee soccer is an adapted sport played by athletes with unilateral lower limb amputations who compete using forearm crutches on a modified pitch. Players perform locomotion, acceleration, and ball contact on a single intact limb while the upper extremities provide propulsion and postural stability through crutch use, a biomechanical context distinct from able-bodied soccer [1]. Participation in amputee soccer has been shown to significantly improve functional performance, body composition, balance, and social integration in individuals with lower limb amputations compared to sedentary counterparts [2,3].

The physical demands of amputee soccer are considerable. Players cover approximately 3000 m per match, with transtibial players demonstrating greater total distances, higher acceleration frequencies, and elevated mean heart rate compared to transfemoral counterparts [4]. Match play results in significant reductions in upper limb and closed kinetic chain performance, underscoring the functional importance of upper extremity capacity in this sport [5]. Sprint and anaerobic performance have been identified as key determinants of competitive success, with body composition and somatotype contributing to performance variance in amputee soccer players [6]. At the elite level, higher vertical jump capacity, trunk flexor strength, and quadriceps endurance differentiate elite from professional-level players, suggesting a clear physical performance gradient within the sport [7]. A recent scoping review identified 29 sport-specific performance tests across 12 studies but concluded that no test has been simultaneously validated, reliable, and standardized for this population [8].

Core stabilization and endurance are fundamental prerequisites for athletic performance, providing the mechanical foundation for force transmission between extremities. In able-bodied football players, trunk muscle endurance has been associated with sprint performance [9]. In amputee soccer, the role of core stabilization may be amplified: with only one intact lower limb and bilateral crutch support, the lumbopelvic region must compensate for the absent contralateral limb contribution to postural control and propulsion. Aytar et al. found a significant correlation between trunk flexor isokinetic strength and dynamic core stability in eleven amputee soccer players [10]. A randomized study demonstrated that core exercise programs produced meaningful improvements in aerobic capacity in amputee soccer players [11]. The relationship between lumbar stabilization capacity measured by pressure biofeedback and physical performance outcomes has not been investigated in this population.

MIP and MEP are indices of respiratory muscle strength, with reduced values associated with impaired exercise capacity in athletic populations [12]. The diaphragm and transversus abdominis share roles in both postural control and intra-abdominal pressure regulation, suggesting that respiratory and core muscle function are interdependent and may jointly influence athletic performance. Inspiratory muscle training improves repeated sprint ability in professional soccer players, with concurrent increases in MIP and peak inspiratory flow [13]. In amputee athletes, amputation-related postural adaptations alter thoracopelvic mechanics, potentially affecting this relationship. To our knowledge, no previous study has examined respiratory muscle strength in relation to physical performance in amputee soccer players.

This study examined associations between core stabilization, core endurance, respiratory muscle strength, isokinetic knee muscle strength, and dominant handgrip strength with physical performance outcomes in amputee soccer players. A secondary aim was to identify the physiological variables most strongly associated with physical performance. We hypothesized that: (1) core stabilization and core endurance would be significantly associated with physical performance outcomes; (2) respiratory muscle strength would be associated with physical performance; (3) isokinetic knee muscle strength of the intact limb would be associated with lower-limb performance outcomes; and (4) dominant handgrip strength would be associated with the upper extremity performance outcome.

## 2. Materials and Methods

### 2.1. Study Design

This study was designed as a descriptive, cross-sectional, and observational study. The study was conducted and reported in accordance with the Strengthening the Reporting of Observational Studies in Epidemiology (STROBE) guidelines.

### 2.2. Participants

#### Sample and Setting

This study was conducted at the Uskudar University Physiotherapy and Rehabilitation Application and Research Center (USFIZYOTEM). Ethics committee approval for the study was obtained from the Uskudar University Non-Interventional Research Ethics Committee at its meeting dated 29 November 2024. Data collection was carried out between January 2025 and March 2025. The study’s scope and purpose were explained to the participants, and they signed an informed consent form. The study was conducted in accordance with the Declaration of Helsinki, and written informed consent was obtained from all participants prior to data collection. Participants were recruited from a professional amputee soccer team competing in the Turkish Amputee Football Super League. Eligible players were required to have a unilateral lower limb amputation, to have been actively playing amputee soccer for at least one year, and to be between 18 and 45 years of age. Players were excluded if they had a bilateral lower limb amputation, had undergone surgical intervention within the preceding six months, presented with neurological, vestibular, or visual impairment, were using medication affecting central nervous system or pulmonary function, or had any musculoskeletal pathology likely to affect motor performance. Of the 20 players initially assessed for eligibility, 15 met the inclusion criteria and were enrolled in the study.

### 2.3. Procedure

Players were recruited through an invitation extended by the club’s management during the off-season period. Data collection was completed in two sessions separated by 48 h. All assessments were performed between 09:00 and 15:00. In the first session, body height was measured using a stadiometer, and body mass was measured using a bioelectrical impedance-based body composition monitor (Omron HBF-511B-E, Omron Healthcare Co., Ltd., Kyoto, Japan), with participants barefoot and in light clothing. Respiratory muscle strength (MIP, MEP), handgrip and pinch strength, and core stabilization (PBU) were subsequently assessed. The triple hop test, sprint test, and CKCUEST were administered using a rotating station format, with a minimum 5-min rest interval provided between successive tests for each participant. Isokinetic knee muscle strength and the McGill Core Endurance Battery were assessed in the second session to avoid fatigue-related interference with the physical performance outcomes assessed in the first session.

### 2.4. Measures

#### 2.4.1. Hand Grip Strength

Dominant handgrip strength was measured with a portable hydraulic hand dynamometer (Jamar; Fabrication Enterprises Inc., New York, NY, USA). Participants were seated with their arm relaxed at the side, elbow flexed to 90°, and the forearm and wrist maintained in a neutral position. Three consecutive measurements were obtained, and the mean value was used for analysis [14].

#### 2.4.2. Isokinetic Knee Muscle Strength

Isokinetic knee muscle strength of the intact limb was assessed using the IsoMed 2000 isokinetic dynamometer (D&R Ferstl GmbH, Hemau, Germany), which has demonstrated excellent reproducibility for isokinetic and isometric maximum knee flexion and extension peak torque measurements (ICC = 0.90–0.98, SEM = 4.0–9.1 Nm) [15]. Testing was performed on the intact limb, as standardized dynamometric testing of the residual limb is not feasible in this population. Participants were seated on the dynamometer chair with their hip at approximately 85° of flexion, and the lateral femoral condyle was aligned with the axis of rotation of the dynamometer. The trunk, pelvis, and thigh were secured with standardized stabilization straps to minimize compensatory movements. Each session began with a familiarization trial prior to data collection, and participants completed a standardized warm-up consisting of five submaximal practice repetitions at each angular velocity. Concentric knee flexion and extension peak torque were then assessed at angular velocities of 60°/s, 180°/s, and 240°/s, consistent with the protocols previously reported in amputee soccer populations [16]. Five maximal repetitions were performed at each angular velocity, with 60 s of passive rest between sets. Verbal encouragement was provided throughout testing. Gravity correction was applied for all measurements. The highest peak torque value obtained across the five repetitions at each angular velocity was recorded in Newton-meters for analysis.

#### 2.4.3. Core Endurance

Core endurance was assessed using the McGill Core Endurance Battery, comprising four isometric tests: the trunk extensor endurance test (Biering–Sørensen test), trunk flexor endurance test, and right and left lateral bridge tests. This battery has demonstrated excellent test–retest reliability in athletic populations, with intraclass correlation coefficients ranging from 0.80 to 0.89 [17]. For the trunk flexor endurance test, participants were seated with their trunk inclined at 60° from the table, the intact hip and knee flexed at 90°, arms folded across their chest, and the intact foot secured with a strap; the trunk support was then removed, and participants maintained the position unsupported. For the trunk extensor endurance test, participants were positioned prone with the upper body unsupported beyond the edge of the table, the pelvis and intact lower limb secured with straps, and the arms folded across the chest. For the lateral bridge tests, participants assumed a side-lying position supported on one forearm and the lateral aspect of the lower body, with the hips lifted to align the trunk in a straight line; on the amputated side, ground contact was provided by the residual limb. Each test was performed from a standardized starting position, and the duration for which the participant maintained correct postural alignment was recorded in seconds. Timing was discontinued when alignment was lost, and the elapsed time was recorded. The sum of all four test durations constituted the McGill total score.

#### 2.4.4. Core Stabilization

Core stabilization capacity was measured using the pressure biofeedback unit (PBU; Chattanooga Group, Hixson, TN, USA), a combined manometer and air-filled pressure cell that detects spinal movement through pressure changes produced by deep abdominal muscle activity, providing an indirect measure of transversus abdominis and lumbar multifidus activation. The device has demonstrated excellent inter-rater reliability for measuring transversus abdominis activity (ICC = 0.94–0.97) [18]. Participants were positioned prone with the PBU placed beneath the abdomen at the level of the anterior superior iliac spine and inflated to a baseline pressure of 70 mmHg. As this test was performed in a prone position without lower-limb loading or standing balance, no protocol modification was required across amputation levels. They were then instructed to perform the abdominal drawing-in maneuver by gently drawing the navel toward the spine, without pelvic movement or breath-holding. The reduction in pressure from the 70 mmHg baseline was recorded in mmHg across three trials, with a 30-s rest interval between trials, and the mean value was used for analysis. In this study, the PBU score represented the magnitude of pressure reduction from the baseline; therefore, lower scores indicated smaller pressure deviation during the maneuver and were interpreted as better lumbopelvic stabilization control [19].

#### 2.4.5. Upper Extremity Functional Performance

Upper extremity functional performance was assessed using the Closed Kinetic Chain Upper Extremity Stability Test (CKCUEST), a field-based measure with reported reliability [20]. Participants assumed a standard push-up position with their hands placed on two parallel lines 91.4 cm apart. From this position, they alternately reached across the body with one hand to touch the opposite hand for 15 s, maintaining trunk stability throughout. The number of touches was recorded as the performance score. Four trials were completed with 45 s of rest between attempts. To reduce potential learning effects, the mean of the final three trials was used for analysis.

#### 2.4.6. Respiratory Muscle Strength

Respiratory muscle strength was assessed using an electronic oral pressure device (Cosmed Pony Fx, Rome, Italy). Maximal inspiratory pressure (MIP) and maximal expiratory pressure (MEP) were recorded. Each measurement was performed three times. Trials were considered acceptable if the difference between attempts did not exceed 10 cmH_2_O or 10%. The highest value was retained for analysis. Reference values for MIP and MEP were determined based on age and sex, and results were expressed as a percentage of predicted values [21,22].

#### 2.4.7. Triple-Hop Test

The Triple-Hop Distance Test (THT) assessed unilateral explosive power of the intact limb. Participants performed three maximal consecutive forward hops on the intact limb from a unipedal stance, with the toe positioned at the starting line and arm swing permitted. Total distance was measured from the starting line to the final heel contact. Up to three practice trials were performed prior to data collection for familiarization. Any trial involving loss of balance or contralateral limb contact before stabilization was repeated. The maximum distance from three successful attempts in athletic footwear was utilized for analysis, following a protocol with high established reliability (ICC = 0.92–0.97, SEM = 4.61–17.74 cm) [23].

#### 2.4.8. Sprint Performance

Sprint performance was assessed over 10, 20, and 30 m using a hand-held stopwatch equipped with a lap/split-time function. Participants started from a stationary standing position behind the start line and performed a maximal 30 m sprint. A single trained assessor recorded split times at 10, 20, and 30 m by pressing the lap button as the participant passed each marker during the same trial. In amputee football players, sprint testing is typically performed using crutches without a prosthesis to reflect sport-specific movement patterns [8]. Three trials were completed with three minutes of passive rest between attempts, and the fastest time at each distance was used for analysis.

### 2.5. Statistical Analysis

Data were analyzed using SPSS 27.0 (IBM Corp., Armonk, NY, USA). Descriptive statistics were expressed as mean ± standard deviation for continuous variables, and as frequency and percentage (%) for categorical variables. Data normality was assessed using the Shapiro–Wilk test and confirmed for all continuous variables. Pearson correlation coefficients were calculated between physiological measures and performance outcomes; variables with significant correlations (*p* < 0.05) were subsequently entered into separate simple linear regression models. The level of statistical significance was set at *p* < 0.05. Given the exploratory nature of this study and the small sample size inherent to this population, no correction for multiple comparisons was applied, and associations should be interpreted accordingly. Post hoc power analysis (G*Power 3.1) for the regression models yielded power estimates of 1.00, 0.72 (f^2^ = 0.51), and 0.68 (f^2^ = 0.46) for Models 1, 2, and 3, respectively; Models 2 and 3 fell below the conventional 0.80 threshold.

## 3. Results

The demographic characteristics of the amputee football players are presented in Table 1. The mean age of the participants was 27.4 ± 5.6 years, with a mean BMI of 20.8 ± 1.4 kg/m^2^. Most of the players had transfemoral amputation (73.3%), and traumatic etiology was the predominant cause (86.7%). The mean duration of sports participation was 6.7 ± 3.2 years. Phantom pain was reported by 33.3% of the participants.

Descriptive statistics for all physiological and physical performance parameters are presented in Table 2. Respiratory muscle strength was 92.04 ± 15.04% and 73.12 ± 11.22% of the predicted values for MIP and MEP, respectively. Intact limb isokinetic knee extension peak torque exceeded the flexion peak torque at all angular velocities tested (60°/s, 180°/s, and 240°/s). The PBU score was 6.20 ± 2.28 mmHg and McGill total score was 268.33 ± 42.22 s. Mean triple-hop test distance was 4.78 ± 0.50 m, 10-m sprint time was 2.35 ± 0.42 s, and the CKCUEST score was 21.93 ± 4.80 touches.

Pearson correlation results are presented in Table 3. The PBU score was negatively correlated with triple-hop test distance (r = −0.928, *p* < 0.001) and 10-m sprint time (r = −0.580, *p* = 0.023). McGill total score was positively correlated with triple-hop test distance (r = 0.561, *p* = 0.030), and dominant handgrip strength was positively correlated with CKCUEST score (r = 0.634, *p* = 0.011). No significant correlations were observed between respiratory muscle strength or isokinetic knee muscle strength and any performance outcome. No significant correlations were found between any physiological variable and the 20- or 30-m sprint times.

Simple linear regression analysis results are presented in Table 4. The PBU score was significantly associated with triple-hop test distance (β = −0.928, R^2^ = 0.861, F(1,13) = 80.8, *p* < 0.001), explaining 86.1% of the variance. In separate models, the PBU score was also significantly associated with 10-m sprint time (β = −0.580, R^2^ = 0.336, F(1,13) = 6.58, *p* = 0.023), explaining 33.6% of the variance, and the McGill total score was significantly associated with triple-hop test distance (β = 0.561, R^2^ = 0.315, F(1,13) = 5.97, *p* = 0.030), explaining 31.5% of the variance. Cook’s distance was calculated for each model to assess influential observations. In Model 1, values ranged from 1.23 × 10^−4^ to 0.620 (Mean = 0.135, SD = 0.209); in Model 2 from 3.25 × 10^−7^ to 0.703 (Mean = 0.101, SD = 0.193); and in Model 3 from 3.63 × 10^−6^ to 0.778 (Mean = 0.100, SD = 0.203). Although the maximum values exceeded the conventional 4/n threshold (0.267) in all three models, all remained below the critical value of 1.0, indicating no undue influence of individual observations on the regression estimates.

## 4. Discussion

This study examined the associations between core stabilization, core endurance, respiratory muscle strength, isokinetic knee muscle strength, and dominant handgrip strength with physical performance outcomes in amputee soccer players. Core stabilization capacity, measured by the pressure biofeedback unit, showed the strongest association with physical performance in amputee soccer players, explaining 86.1% of the variance in triple-hop test distance. PBU score was also associated with 10-m sprint time. Core endurance assessed by McGill total score was also associated with hop test distance, and dominant handgrip strength was positively correlated with CKCUEST performance. Respiratory muscle strength and isokinetic knee muscle strength showed no significant associations with any performance outcome. To our knowledge, this is the first study to examine the relationship between PBU-based lumbar stabilization and physical performance in amputee soccer players, and the first to report MIP and MEP values in this population.

The strong negative association between PBU score and triple-hop test distance, where lower pressure deviation reflects better lumbar stabilization and corresponds to greater unilateral explosive capacity, represents the primary finding of this study. In amputee soccer, all propulsive and deceleration demands are channeled through a single intact limb and forearm crutches, placing substantial demands on lumbopelvic control for lower extremity force production [1]. Core stability has been defined as the capacity to control trunk position and movement for optimal force production, transfer, and control between the extremities and the environment [24]. The lumbopelvic region functions as a proximal stabilizing platform from which distal segments generate and transmit force, and insufficient proximal control is expected to reduce distal output [25]. Core strength and neuromuscular control have been identified as integral components of athlete performance across multiple sport contexts [26]. Aytar et al. previously found a significant correlation between trunk flexor isokinetic strength and dynamic core stability in eleven amputee soccer players, establishing that core function is relevant to performance in this population [10]. The present findings extend this observation by showing that deep stabilization capacity, measured by PBU, explains 86.1% of the variance in hop test performance. This proportion is markedly larger than those typically observed in able-bodied athletes, where core stability measures have shown weak to moderate associations with physical performance outcomes [9,27]. This disparity may reflect the amplified mechanical demands placed on the lumbopelvic region during unilateral single-limb loading, consistent with biomechanical evidence showing increased trunk muscle loading and lateral trunk compensation toward the residual side during single-limb stance in individuals with lower-limb amputation [28]. Core stabilization training improves triple-hop limb symmetry in athletes [29], and core muscle fatigue reduces lower extremity stiffness during hopping by 15–16% in healthy basketball players [30]. These findings further support the mechanical pathway through which core function influences explosive performance. PBU score was also associated with 10-m sprint time (R^2^ = 0.336); however, the direction of this association was unexpected, as higher PBU values, indicating poorer stabilization, were associated with faster sprint times. Given the small sample size, this finding should be interpreted with caution and warrants further investigation in larger cohorts.

McGill total score was significantly associated with triple-hop test distance in a separate simple regression model (R^2^ = 0.315, *p* = 0.030), explaining 31.5% of the variance and indicating that isometric trunk endurance is also relevant to unilateral explosive performance. In lower limb amputees, reduced trunk extensor endurance has been associated with worse physical performance across multiple functional outcomes [31], and amputee soccer players demonstrate markedly greater back extensor endurance than sedentary amputees, alongside superior balance, agility, and anaerobic power [3]. McGill total score has been associated with vertical jump and sprint performance in collegiate football players [9], and with upper extremity functional performance in collegiate athletes [32]. The moderate negative correlation between PBU score and McGill total score (r = −0.636, *p* = 0.011) suggests that these constructs are partially distinct, sharing approximately 40% of common variance. This is consistent with theoretical frameworks distinguishing neuromuscular control of deep stabilizers from global trunk endurance [33], and with empirical evidence showing that lumbopelvic motor control assessed by pressure biofeedback is not associated with core strength or endurance measures [34]. These measures may therefore reflect partially distinct aspects of core function; however, because each was examined in a separate simple regression model, their unique contributions to hop test performance cannot be determined without multivariable analysis. Comprehensive core assessment in amputee soccer may nonetheless benefit from addressing both stabilization control and endurance capacity.

Dominant handgrip strength was significantly associated with CKCUEST score (r = 0.634, *p* = 0.011). In amputee soccer, forearm crutches serve both propulsive and stabilizing functions throughout match play, placing continuous demands on upper extremity musculature [35]. Post-match reductions in upper extremity performance have been documented in amputee soccer, reflecting the functional importance of this capacity [5]. Handgrip strength was not associated with sprint performance, consistent with Wieczorek et al., who found no relationship between handgrip strength and sprint times in thirteen amputee soccer players [36]. This pattern suggests that handgrip strength is more relevant to closed kinetic chain upper extremity stability than to linear speed; sprint performance in this population is more specifically predicted by shoulder extension and latissimus dorsi strength [35], which are directly engaged in crutch propulsion mechanics.

Neither MIP nor MEP was significantly correlated with any performance outcome. Although inspiratory muscle training improves repeated sprint performance in able-bodied soccer players [13], resting respiratory muscle strength shows only modest correlations with exercise capacity in trained athletes [37]. This suggests that baseline values may not be performance-limiting in competitive populations. The intermittent nature of amputee soccer may further reduce the relative contribution of respiratory muscle strength to field-based performance. The lower MEP values relative to MIP observed in this sample (73.12 ± 11.22% vs. 92.04 ± 15.04% of predicted) may reflect altered trunk muscle recruitment patterns associated with unilateral amputation. Transtibial amputees have been shown to exhibit asymmetric external oblique activation during postural tasks, with greater recruitment on the intact side compared to controls [38]. Abdominal muscle force-generating capacity is the primary determinant of MEP [39]. It may therefore be hypothesized that chronic postural demands associated with unilateral amputation alter trunk muscle recruitment patterns in ways that reduce expiratory force capacity. However, the present study did not include electromyographic or trunk activation measures, and this proposed mechanism remains speculative. Future studies incorporating the direct assessment of trunk neuromuscular activity alongside respiratory muscle testing are needed to evaluate this hypothesis.

Isokinetic knee muscle strength of the intact limb was not significantly associated with any performance outcome. This finding may reflect the biomechanical specificity of crutch-assisted locomotion, in which propulsive demands are distributed across both upper and lower extremities in a pattern distinct from able-bodied sprinting or hopping [1]. This redistribution is consistent with biomechanical evidence showing that double-crutch use transfers a substantial share of propulsive load to the upper extremities, unlike single-crutch use, which concentrates load on the intact limb [40]. Isolated isokinetic peak torque may therefore not adequately reflect the neuromuscular demands of performance tasks in this population. The predominance of transfemoral amputees in this sample (73.3%) may also be relevant. Transfemoral amputees demonstrate more pronounced gait asymmetry, greater mediolateral center of pressure displacement, and higher stabilization demands compared to transtibial amputees [41], and both transfemoral and transtibial amputees rely on lateral trunk flexion toward the intact side as a compensatory gait strategy [42]. In transfemoral-dominant samples, lumbopelvic stability may therefore exert a stronger influence on functional performance than isolated knee extensor strength.

Several limitations should be acknowledged. The cross-sectional design precludes causal inference. Sprint performance was assessed using a hand-held stopwatch rather than timing gates or photocell systems, which may introduce measurement error; the intra-rater consistency of manual timing was not formally assessed. The sample was recruited from a single team, which may limit generalizability. Player position was not controlled for in the analyses; positional demands differ substantially in amputee soccer and may represent an additional source of variance in performance outcomes. Future studies should consider stratifying analyses by amputation level and player position. The correlations examined across multiple performance outcomes were not adjusted for multiple testing; therefore, some of the statistically significant associations reported may represent false-positive findings. The small sample size, while consistent with the broader amputee soccer literature, limits statistical power and warrants replication in larger cohorts.

## 5. Conclusions

This study identifies core stabilization, assessed by the pressure biofeedback unit, as the dominant physiological factor most strongly associated with physical performance in amputee soccer players, explaining 86.1% of the variance in unilateral hop distance. Core endurance and dominant handgrip strength were also associated with hop test and upper extremity stability performance, respectively, while respiratory muscle strength and isokinetic knee strength showed no significant association with performance outcomes in this population. Given the exploratory nature of this study, the small sample size, and the absence of correction for multiple comparisons, these findings should be regarded as preliminary. Lumbopelvic stabilization and endurance may warrant attention in assessment and training programs, but firm practical recommendations require confirmation in larger, adequately powered samples. Longitudinal studies incorporating larger, multi-team cohorts are also needed to establish the directionality of these associations and to evaluate whether targeted core training improves on-field performance.

## Figures and Tables

**Table 1:** Demographic and clinical characteristics of the amputee soccer players.

Characteristics		Total Amputee Players (*n* = 15)
Age (years)		27.4 ± 5.6
Height (cm)		170.9 ± 6.5
Weight (kg)		60.7 ± 6.1
BMI (kg/m^2^)		20.8 ± 1.4
Amputation side *n* (%)		Left: 11 (73.3%), Right: 4 (26.7%)
Dominant side *n* (%)		Left: 6 (40.0%), Right: 9 (60.0%)
Age at amputation		10.5 ± 4.9
Years of playing experience		6.7 ± 3.2
Phantom pain *n* (%)		Yes: 5 (33.3%), No: 10 (66.7%)
Phantom pain severity (VAS, *n* = 5)		1.6 ± 1.9
Amputation level *n* (%)	Transfemoral	11 (73.3%) (Left: 8, Right: 3)
	Transtibial	4 (26.7%) (Left: 3, Right: 1)
Amputation cause *n* (%)	Congenital	1 (6.7%)
	Traumatic	13 (86.7%)
	Tumoral	1 (6.7%)
Playing position	Stoper	4 (26.7%)
	Midfielder	3 (20.0%)
	Winger	5 (33.3%)
	Forward	3 (20.0%)

Values are presented as mean ± standard deviation (SD) or number (percentage), as appropriate. BMI: body mass index; VAS: Visual Analog Scale.

**Table 2:** Physiological and physical performance characteristics of amputee soccer players.

Characteristics	Total Amputee Players (*n* = 15)
*Respiratory Muscle Strength*	
MIP (cmH_2_O)	100.31 ± 15.63
MIP (cmH_2_O) Pred	109.15 ± 2.20
MIP %	92.04 ± 15.04
MEP (cmH_2_O)	110.89 ± 15.44
MEP (cmH_2_O) Pred	152.03 ± 4.45
MEP %	73.12 ± 11.22
*Peripheral Muscle Strength*	
Handgrip Dominant (kg)	48.87 ± 9.83
Handgrip Non-Dominant (kg)	46.05 ± 9.40
Dominant Pinch (kg)	7.31 ± 2.17
Non-Dominant Pinch (kg)	7.15 ± 2.21
*Isokinetic Knee Muscle Strength*	
Flexion peak torque 60° (Nm)	112.00 ± 17.93
Extension peak torque 60° (Nm)	143.73 ± 24.70
Flexion peak torque 180° (Nm)	101.27 ± 17.33
Extension peak torque 180° (Nm)	127.73 ± 23.76
Flexion peak torque 240° (Nm)	88.80 ± 15.67
Extension peak torque 240° (Nm)	112.00 ± 20.91
*Core Stabilization Parameters*	
PBU	6.20 ± 2.28
Trunk Extension Test (s)	66.43 ± 15.26
Trunk Flexion Test (s)	77.47 ± 14.23
Right Lateral Bridge Test (s)	62.74 ± 9.88
Left Lateral Bridge Test (s)	61.69 ± 15.87
McGill Total Score	268.33 ± 42.22
*Physical Performance Parameters*	
10 m sprint (s)	2.35 ± 0.42
20 m sprint (s)	4.50 ± 1.21
30 m sprint (s)	5.45 ± 1.28
THT (m)	4.78 ± 0.50
CKCUEST (average touch)	21.93 ± 4.80

Values are presented as mean ± standard deviation (SD) or number (percentage), as appropriate. n: number of players. MIP: maximal inspiratory pressure, MEP: maximal expiratory pressure, PBU: pressure biofeedback unit, THT: triple-hop test, CKCUEST: Closed Kinetic Chain Upper Extremity Stability Test. Isokinetic measurements were performed on the intact limb at angular velocities of 60°/s, 180°/s, and 240°/s.

**Table 3:** Pearson correlation coefficients between physiological factors and physical performance outcomes.

	Sprint 10 m	Sprint 20 m	Sprint 30 m	THT	CKCUEST
PBU	r = −0.580 *	r = −0.513	r = −0.438	r = −0.928 ***	r = −0.305
McGill Total Score	r = 0.202	r = 0.249	r = 0.180	r = 0.561 *	r = 0.447
MIP	r = 0.215	r = 0.316	r = 0.341	r = 0.310	r = 0.087
MEP	r = 0.227	r = 0.379	r = 0.469	r = 0.373	r = −0.072
Extension Peak Torque 60°/s (Nm)	r = −0.472	r = −0.371	r = −0.427	r = −0.447	r = 0.085
Flexion Peak Torque 60°/s (Nm)	r = −0.506	r = −0.358	r = −0.366	r = −0.455	r = −0.177
Extension Peak Torque 180°/s (Nm)	r = −0.479	r = −0.354	r = −0.383	r = −0.503	r = 0.112
Flexion Peak Torque 180°/s (Nm)	r = 0.095	r = 0.146	r = 0.141	r = −0.327	r = −0.375
Extension Peak Torque 240°/s (Nm)	r = 0.109	r = −0.154	r = −0.083	r = −0.073	r = −0.277
Flexion Peak Torque 240°/s (Nm)	r = 0.060	r = −0.163	r = −0.164	r = 0.009	r = −0.214
Dominant Handgrip Strength (kg)	r = 0.114	r = 0.230	r = 0.247	r = 0.318	r = 0.634 *

* *p* < 0.05, *** *p* < 0.001. PBU: pressure biofeedback unit, MIP: maximal inspiratory pressure, MEP: maximal expiratory pressure, THT: triple-hop test, CKCUEST: Closed Kinetic Chain Upper Extremity Stability Test. Isokinetic measurements were performed on the intact limb. Values represent Pearson correlation coefficients (r).

**Table 4:** Separate simple linear regression models for physical performance outcomes in amputee soccer players.

	Model 1	Model 2	Model 3
Dependent Variable	THT (m)	Sprint 10 m (s)	THT (m)
Predictor	PBU	PBU	Total McGill Score
B	−0.206	−0.108	0.007
SE	0.023	0.042	0.003
β	−0.928	−0.580	0.561
95% CI for B	[−0.255, −0.156]	[−0.198, −0.017]	[0.001, 0.013]
t	−8.99	−2.57	2.44
R^2^	0.861	0.336	0.315
Adjusted R^2^	0.851	0.285	0.262
F(1,13)	80.8	6.58	5.97
*p*	<0.001 *	0.023 *	0.030 *
Cook’s D (Max)	0.620	0.703	0.778

B: unstandardized regression coefficient; SE: standard error; β: standardized regression coefficient; CI: confidence interval; PBU: pressure biofeedback unit; THT: triple-hop test. Separate simple linear regression models were constructed for each predictor demonstrating a significant bivariate correlation with performance outcomes (* *p* < 0.05).

## Data Availability

The data presented in this study are available on request from the corresponding author. The data are not publicly available due to privacy restrictions, as participant informed consent, in accordance with the Turkish Personal Data Protection Law (KVKK, Law No. 6698), covered the use of data for the present study only and did not extend to public deposition in an open-access repository. The dataset can be made available to the editor or reviewers for verification upon request.

## Abbreviations

The following abbreviations are used in this manuscript:

PBU	Pressure Biofeedback Unit
THT	Triple-Hop Test
CKCUEST	Closed Kinetic Chain Upper Extremity Stability Test
MIP	Maximal Inspiratory Pressure
MEP	Maximal Expiratory Pressure
BMI	Body Mass Index
VAS	Visual Analog Scale
ICC	Intraclass Correlation Coefficient
